# Work-Family Conflict Impact on Psychological Safety and Psychological Well-Being: A Job Performance Model

**DOI:** 10.3389/fpsyg.2020.00475

**Published:** 2020-03-31

**Authors:** Bojan Obrenovic, Du Jianguo, Akmal Khudaykulov, Muhammad Aamir Shafique Khan

**Affiliations:** ^1^School of Management, Jiangsu University, Zhenjiang, China; ^2^School of Management, Wuhan University of Technology, Wuhan, China

**Keywords:** work-family conflict, psychological well-being, psychological safety, job performance, work conflict, family conflict

## Abstract

In a modern working environment characterized by new technology and work assignments extended to personal time, employees are expected to balance multiple roles while maintaining maximum productivity. Past studies analyzed work-family conflict and its connection to job performance, without adequate integration of psychological factors into the research model. This study aims to fill the gap and explain the impact of work-family conflict and psychological factors on job performance. To explore the association between work-family conflict and job performance and measure the effects on psychological safety and psychological well-being, an empirical study was conducted on a sample of 277 company employees in Bahrain. The online questionnaire used five-point Likert-scales adopted from previous studies to measure the variables of the research model. In the structural model, relationships between work-family conflict, psychological well-being, psychological safety, and job performance were tested. Confirmatory Factor Analysis with Maximum likelihood estimation was performed by using SEM software AMOS version 23. The findings of the study suggest there is a negative impact of work-family conflict on psychological safety and psychological well-being. This study is significant since it detaches from the prior researches focused on observing the repercussions of work-family conflict in workers’ well-being, and centers on the analysis of job performance instead. The findings show that psychological well-being and psychological safety influence job performance. When psychological well-being and psychological safety of employees are unsatisfactory, job performance will decrease accordingly. The mediation test indicated that work-family conflict had an indirect influence on job performance when psychological safety and psychological well-being were mediators. The study contributes to a better understanding of work-family conflict, psychology of employees, and job performance. The study provides valuable insight to organizations on ways to increase employees’ effectiveness and ensure better performance by preventing work-family conflict from occurring.

## Introduction

Work-family balance is hard to sustain in modern industrial societies due to increasing demands at work and in family settings. Individuals are asked to manage multiple roles simultaneously, allocating their resources between work and family (Fotiadis et al., 2019). Work-family conflict is a psychological phenomenon of imbalance between work and home life (Csikszentmihalyi, 2003). The most common stressors conducive to occurrence of work-family conflict are job burnout, dissatisfaction, work stress, long working hours, and role conflict (Kossek and Ozeki, 1998; Spector et al., 2004; Bakker et al., 2005; Ford et al., 2007). Work overload and stressful events caused by the work environment (Cartwright and Pappas, 2008; Ganster and Perrewé, 2011) may physically and emotionally exhaust an employee in a way that it gives rise to work-family conflict (Frone et al., 1997b; Baeriswyl et al., 2016). The two-way model of work-family conflict shows that stress factors in the workplace such as lack of autonomy or excessive workload have a negative impact on the “work” side, whereas stress factors related to family such as misbehaving children or overly dependent parents harm the “family” side of the balance (Liu et al., 2019). In other respects, excellent compatibility between family and work gives a sense of high achievement in the workplace since it motivates individuals (Baeriswyl et al., 2016).

In literature, the potential impact of work-family conflict on organizational and personal well-being has been discussed (Kossek and Ozeki, 1998; Ford et al., 2007). Work-family conflict hurts employees’ productivity and harms job performance by decreasing the job satisfaction of employees (Johnson et al., 2005). It also affects employee turnover, psychological distress, and life satisfaction (Greenhaus and Beutell, 1985).

A considerable amount of organizational effort has been directed into the research of the destructive consequences of work-family conflict (Grant-Vallone and Donaldson, 2001; O’Driscoll et al., 2004). Attrition of staff and other occupational threats can have negative implications for the performance of organizations. On the other hand, job performance, and mental health of individuals increase when the organizational principles encourage work-family balance (Fitzpatrick et al., 2012). Knowing that a “happy” employee is a more productive employee, organizations become increasingly interested in the family life of employees and engage actively in resolving work-family conflicts. Here it becomes essential to consider factors generating psychological contentment and thus inciting more significant work achievements. At the same time, factors that bring about uncertainty, dissatisfaction, insecurity, and conflict are responsible for decreasing personal as well as organizational well-being and therefore warrant more attention. A limitation of prior studies in the psychological well-being context is that they do not sufficiently explore impact of non-organizational factors on performance. This study addresses the limitation by introducing family as a key factor that has implications for the well-being and psychological safety of individuals. Up until this point, there has been very little empirical investigation on the relationship between work-family conflict and work performance. Past research has mostly focused on limiting the effect of work-family conflict on few variables, such as leadership (Major and Cleveland, 2007; Hammer et al., 2009; Major and Morganson, 2011,; Matthews et al., 2013; Hill et al., 2016), organizational support (Keoboualapheth et al., 2017), psychological well-being (e.g., Allen et al., 2000; O’Driscoll et al., 2004; Ibrahim et al., 2009; Karimi et al., 2011; Lee et al., 2013; Matthews et al., 2014), psychological safety (Cullen, 2005; Dollard and Bakker, 2010; Dollard and Karasek, 2010; Hall et al., 2010; Murphy, 2011), and the relation between work-family conflict and work-family relationship with work stress (Kazmi et al., 2017; Lu et al., 2017; Smith et al., 2018), exhaustion (Chen and Huang, 2016), burnout (Montgomery et al., 2003), job control (Proost et al., 2010; Golden et al., 2013), job satisfaction (Aryee et al., 2005; Ford et al., 2007; Kafetsios, 2007) and turnover intention (Lu et al., 2017). Despite research advances, work-family scholars still lack clear understanding of how constructs of the psychological well-being and psychological safety relate to employee’s job performance Resolution of work-family conflict affects employee psychology that translates to positive work behavior.

We asses psychological factors of well-being and safety in a collectivistic society. Up to date majority of studies were conducted in predominately individualistic societies in Western countries, and to our knowledge, this research is significant in that it is first of its kind to have examined well-observed phenomena in the new untapped territory in the context of Bahrain. The empirical study of employees from Islamic culture is a major innovation of the study. The spillover effect of work-family conflict is found to be different in societies with strong tradition of marital commitment (Liu et al., 2016), such are the cultures that should be understood in light of family obligations, where workers are more prone to feel exhausted and less able to cope with stress (Brough and O’Driscoll, 2005; Sun and Pan, 2008).

We illustrate this argument by conducting an analysis building on the Conservation of Resources (COR) theory by identifying important characteristics that should be considered as precedents of work-family conflict (Hofstede, 2003). COR theory can be applied to examine how personal engagement with work assignments and psychological safety can be related to stress and work-family conflict, in organizational settings (Hobfoll, 2004; Chen and Huang, 2016) and in mental health care organizations (van Woerkom et al., 2016). The COR theory has previously been applied to job burnout, work-overload, and loss of organizational and human resources. Building on the theory helps investigate how colleague support and self-esteem, antecedents of psychological safety, can serve as predictors for personal engagement. That is associated with providing a psychologically safe environment as a mean to prevent work-family conflict (Morgeson et al., 2017). Employees of higher well-being display better psychological resources, e.g., they appear more optimistic, resilient while experiencing a setback and have a greater capacity to cope with issues. Moreover, in their survey Costa et al. (2015) found performance feedback, social support from co-workers and supervisors act as job resources predicting work engagement and performance output.

Given that the Job Demands-Resources (JD-R) theory has been recognized as one of the leading frameworks to investigate the factors related to psychological well-being and optimal organizational functioning, we also extended the JD-R theory. The study highlights work-family conflict stressors and suggests it is directly linked to employee psychological well-being, psychological safety, which are, in turn, related to job performance. The focal objective of this study is to explain the relationship between work-family conflict and job performance. By means of introducing factors of psychological well-being and psychological safety into the job performance model, organizational literature gaps are addressed, and empirical evidence is generated in the context of a company in Bahrain. In addition, there remains a need to examine the process through which psychological well-being and psychological safety in the presence of work-family conflict decrease the level of job performance. Toward this end, the current study extends the existing literature by testing mediating effects wherein we seek to explain how interaction of work and family domain impacts employee performance. Future research should be directed toward assessing the same hypotheses and validating our findings in different context.

Innovative points of the study translate to practical implications for managers and leaders, suggesting they must not only inspire and foster a healthy work climate that will motivate employees, but also alleviate work-family conflict. The current study contributes to the existing occupational psychological health and managerial literature, also suggesting for future studies to consider other possible determinants, such as strong traditional and religious values, that may, through certain working outcomes, yield work-family conflict.

## Theory and Model

Job Demands-Resources theory, demands-control model, the person-environment fit approach and COR theory are influential job-related stress theories that have had a prominent impact on the field of work and well-being related psychology (Bakker and Demerouti, 2007, 2014, 2018). JD-R theory investigates the impact of working conditions on employees and the impact employees have on the working conditions. According to the JD-R theory factors of well-being and organizational behavior influence each other over time on organizational, team, and individual levels (Bakker and Demerouti, 2018). JD-R theory outlines the processes where work requirements may impact job health, well-being, organizational behavior, and job performance. More precisely, job stressors are identified on the basis of self-reported states and perceptions of the individual employees. When organizational leaders know which particular tasks and problems require immediate attention, strategic, and structural measures are introduced in order to optimize problematic processes for the employees to allow them to improve the quality of their work life. In a similar vein, family issues can be characterized as the disturbances affecting job performance. We explore the existing theoretical background of COR theory and prior research that applied it (Hobfoll, 2004) to examine work and stress in an organizational setting and in mental health care organizations (Westman et al., 2004; van Woerkom et al., 2016). The study of Chen and Huang (2016) conducted on the sample of employees from R&D departments in the IT technology industry in China utilized COR to examine how personal engagement is related to work-family conflict.

A study by Halbesleben et al. (2014) proposed COR as a theory of motivation with the basic tenet that humans are motivated to protect their current resources and acquire new ones. We argue that this directly concerns well-being. COR theory is a stress and motivational theory, a proposed explanatory model for investigating how employees are to be affected by stressful circumstances, identifying those circumstances, and speculating on how individuals cooperate in order to cope with them. Application of COR theory was previously found to be related to job burnout, work-overload, and loss of organizational and human resources. Using the JD-R framework helps to understand how perceived organizational support can mitigate the impact of job demands on workload and emotional demands, thus decreasing absenteeism from work. We argue that providing the holistic organizational support to employees while balancing their family and job demands may be a key tool for organizations to reduce the dispersion or even loss of valuable resources by adjusting or redesigning job demands.

### Work-Family Conflict Impact on Psychological Well-Being and Psychological Safety

Psychological well-being is a broad concept that captures emotional and mental conditions, level of satisfaction from work, and overall life satisfaction. An individual’s overall effectiveness in terms of psychological functioning is defined by psychological well-being, which is primarily used to measure hedonic and satisfaction levels (Cartwright and Pappas, 2008). A sense of control over work and family activities promotes psychological well-being (Fitzpatrick et al., 2012). A sense of control can be characterized by the time individuals perceive as available to fulfill their role-related requirements and, as such, constitutes work-family balance. Fotiadis et al. (2019) state that work-life conflict leads to decreased employee well-being and increased psychological stress. There is a relationship between work-related pressure and psychological health. For instance, moderate work pressure makes employees develop professionally by accepting the challenge, resulting in good psychological health (Harter et al., 2002). Negative work-family interaction decreases well-being due to depleting mental resources and high psychological strain (Grandey and Cropanzano, 1999; Voydanoff, 2002; Eby et al., 2005).

Conservation of Resources theory provides an integrated theoretical framework that helps to elucidate both conflicting and enriching dynamics of resource investment processes, namely, the interaction of work-family conflict and work-family enrichment, by posing them in a common resource-exchange economy (Grandey and Cropanzano, 1999; McNall et al., 2010; Hobfoll et al., 2018). Through the lens of a basic tenet of COR, work and family domains are accounted for as reservoir of resources, where a potential or actual threat, loss or a gain in one domain affects the basic state of the other (Hobfoll, 2002). Stress in managing multiple roles manifests as work-family conflict when the demands in one domain hinder the expectations of the other, thus causing strain, depriving the individual of valuable energy and forcing him to invest more psychological and physiological resources in the problematic realm. Each time more conflict is experienced in one domain, fewer resources are available to fulfill one’s role in another. In the process of juggling work and family roles, resources are lost, thus triggering a diminished or a negative state of being – conflict. Correspondingly, adding to COR theory regarding the loss of resources employees who had lost their enthusiasm and showed more burnout complaints reported a stronger increase in work overload, work hours, and work-family barriers (ten Brummelhuis et al., 2011). Additionally, there is a significant relationship between work-family conflict, personal burnout and distress symptoms (Noor, 2002).

In a study of Ibrahim et al. (2009) work-family conflict was the main factor influencing employee well-being in South-East Asian countries due to the change in workforce demographics and the increasing female participation. Work-family conflict has escalated due to the higher participation of women in the workforce (O’Driscoll et al., 2004), changing family role expectations, and technological developments, along with greater expectation on individuals to “work anytime, anywhere,” thus inducing the psychological strain and reflecting negatively on employees’ psychological well-being. Panatik et al. (2011) conducted a study on 100 schoolteachers in Malaysia, identifying several work-family conflict specific determinants influencing psychological well-being. They found work-family conflict manifests in the increased levels of anger and aggression, thus contributing to mental health problems and causing workers to quit their jobs.

From the above presented evidence, we conclude that work-family conflict is a significant predictor of employee psychological well-being.

Accordingly, we hypothesize:

**Hypothesis 1a:** Work-family conflict has a negative impact on psychological well-being.

Work-family relationship contributes to the creation of psychological climate at work, whereas a favorable psychological climate creates the perception of psychological safety within the organization (Cartwright and Pappas, 2008). The organization is considered to have a psychologically safe climate when an employee feels that the working environment complements his well-being (Faragher et al., 2004). In the absence of favorable psychological climate, the psychological safety level is likely to be low. Work-family conflict has also been linked to mental health problems, and it may lead to a decline in an individual’s mental abilities (Zapf et al., 1996). When individuals’ work-stress is reflected in their relationship with family members, reducing the effectiveness of their work and family roles may harm their psychological well-being and psychological safety. Psychological safety is a critical psychological circumstance that “shapes how people inhabited their roles [in the organization]” (Kahn, 1990). Psychological safety embodies a feeling that employees can “show and employ one’s self without fear of negative consequences to self-image, status, or career” (Kahn, 1990).

According to recent studies and extensions on COR theory, resource loss directly predicted the occurrence of work-family conflict, while resource gain weakened the positive relationship between work-family conflict (Chen and Powell, 2012; Chen et al., 2014). Conflict is therefore related to the downward change in resources (Oren and Levin, 2017). Stressful situations disrupt employee’s perception of psychological safety as key resources are found to be endangered. Stress is elicited with each threat to loss of resources, such as economic uncertainty, the threat to self-esteem, fear for one’s marital status or job security, financial downfall or a sick relative (Hobfoll, 1991, 2001; Hunter and Csikszentmihalyi, 2003; Reizer et al., 2010). Married status and tenure are depicted by this theory valued and sought resources considering that those who are married have more influx to draw on, such as emotional support, empathy and finances, while tenure ensures greater performance and increased psychological safety. For instance, when a partner’s resources at work increase, the other spouse may provide encouragement and contribute to the latter’s well-being. COR model was found to have a suitable explanation for both intra-and inter-role stress, where job and family distress are mutually related, e.g., trouble in one domain leads to problems in other (Grandey and Cropanzano, 1999). Furthermore, according to the crossover model (Westman, 2001), psychological stress and work-family conflict may transmit via the spillover effect on the other team members. These negative experiences are related to a desire to minimize the loss of resources, which may have a less favorable result for the organization, such as quitting the job or filing for sick leave. Work-life interaction is a significant factor affecting employee life quality, and it can influence individual satisfaction, safety, and health (Grant-Vallone and Donaldson, 2001). Work-family conflict occasionally has detrimental consequences such as depression (Higgins et al., 1992; Frone et al., 1997a, b), and alcohol abuse (Frone et al., 1997a). There is also a positive effect of reduced work-family conflict, leading to greater work engagement and personal satisfaction (Grant-Vallone and Donaldson, 2001; Robertson et al., 2012), factors conducive to psychological safety.

Accordingly, we derive the following hypothesis:

**Hypothesis 1b:** Work-family conflict has a negative impact on psychological safety.

### Psychological Well-Being and Job Performance

Job performance can be defined as an individual’s effort to fulfill workplace responsibilities. According to Johnson et al. (2005), 25% of employees in the United Kingdom relate job performance to psychological well-being. The COR theory follows an understanding that individuals strive to obtain, retain, foster, and protect things they centrally value, therefore, their psychological well-being will accordingly be highly dependent upon the influx and retention of key resources, such as marital support and professional satisfaction. According to the COR theory, employees will engage in behaviors to avoid the negative impact of resource loss on well-being (Whitman et al., 2014). Academics strived for years to investigate different work-family resource investment processes and their impact on job performance and satisfaction (Jansen et al., 2003; Innstrand et al., 2008; Langballe et al., 2011; Gao et al., 2013; Liu et al., 2016; Goldfarb and Ben-Zur, 2017).

One of the core objectives of this research is to question to what extent psychological well-being is different from merely positive job attitudes and engagement and to uncover to which extent it can be used to explain variance in job performance. The task at hand was already the focal point of some prior works, aiming to predict the performance by investigating either positive work attitudes, namely engagement or well-being. For instance, Harrison et al. (2006) proposed a unified attitude-engagement model where positive employee attitudes, such as job satisfaction and commitment, were found to be associated with better performance. In their study, the construct of positive work attitudes included items centering around job satisfaction, organizational citizenship, and organizational attachment. They’ve managed to relate positive work attitudes to performance and got the attention of senior managers due to the enticement of research evidence connecting them with enhanced productivity (Harter et al., 2002; Towers Perrin, 2007). Psychological well-being should be grounded in its relation to positive work attitudes to the extent that it can explicitly be stated that psychological well-being is associated with performance, engagement and related job attitudes. On the other hand, psychological well-being is also explored individually (Bakker, 2009). A positive experience is linked to improved psychological and physical health. According to Cartwright and Cooper (2008), individuals with higher levels of psychological well-being are healthier and more productive at work (Wright and Cropanzano, 2000).

It seems the role of psychological well-being is more prominent in causing rather than predicting variance in performance. Employees with higher well-being display better psychological resources, are more optimistic, resilient and have a greater capacity to cope with issues. High levels of psychological well-being are strongly associated with numerous positive aspects in terms of personal life and professional career (Lyubomorsky et al., 2005). To fully grasp this concept, it must be approached holistically and not only contextually. Although it’s not a purely context-dependent phenomenon, it can be influenced by environmental, organizational, and societal events and thus subject to therapeutic interventions.

In line with fundamental tenets of COR, social environment and context may influence how resources are nurtured and maintained, with emphasis on the critical role of social support, be it a supportive family or organization. Previous analysis already established the link between self-esteem, optimism, and self-efficacy emerging from common developmental conditions (Hobfoll et al., 2018). Likewise, employees’ psychological well-being will positively influence job performance. For instance, evidence shows a positive leader-follower exchange is related to job resources facilitating job performance (Breevaart et al., 2014). Furthermore, a bulk of academic effort building on COR explored how emotional exhaustion affected resource investment strategies tied to performance at work (Wright and Cropanzano, 1998; Halbesleben and Bowler, 2007; Demerouti et al., 2014; Park et al., 2014). Some studies aimed to identify resources conductive to improving occupational well-being. Findings determined mentoring programs and social events, providing job candidates with realistic information and social activities to be especially useful to employees’ psychological well-being (Kiazad et al., 2014).

Existing literature also indicates that psychological well-being impacts employees’ job performance (Wright and Cropanzano, 2000; Bakker and Schaufeli, 2008). Higher psychological well-being is associated with higher employee effectiveness (Wright et al., 1993) and productivity (Carvalho et al., 2018). Participants with higher well-being are superior decision-makers with greater interpersonal behaviors and higher performance ratings (Wright and Cropanzano, 2004). In order to meet desired targets and produce projected outputs, employees are required to be psychologically fit, implying that their mental focus should solely be directed toward work tasks. As soon as a distraction rooted in instability and lack of well-being is present, the focus shifts from the work task to the personal issue. As a result, employee productivity decreases. The outputs are often of inferior quality, and the delays in operations may arise.

Psychological well-being also has a positive impact on an employee’s learning attitude (Edmondson, 1997). Subsequently, a willingness to learn in a workplace can create a favorable environment for employee performance. In recent years organizations are increasingly taking on responsibility through investment in supportive social policies, acting as an organization-based resource of support and a buffer against the resource-depleting effect of high workload (van Woerkom et al., 2016). Employees are given coping strategies in order to increase their resilience to stressful situations. When employees believe their organization values their contributions, they put a significant amount of energy to improve their job performance. Moreover, in line with Westman (2001) crossover theory, emotional and positive experiences spread from one co-worker to another, thus leading to an upward spiral by influencing the team, the department and the organization. Conversely, lack of organizational improvements for the mental well-being of employees causes employees to be stressed and unproductive (Carvalho et al., 2018). Lack of psychological well-being of employees may cause damage to their physical health. Depression and loss of self-esteem have been linked both to poor work performance (Quick et al., 1997) and to dysfunctional psychological well-being (Ivancevich and Matteson, 1980). Correspondingly, due to absence from work, employees’ work performance declines, and organizations incur financial losses. Current theory suggests that psychological well-being or lack thereof has a significant impact on overall job performance.

Based on the related arguments of psychological well-being, we infer the hypothesis as follows:

**Hypothesis 2:** Psychological well-being impacts job performance.

### Psychological Safety and Job Performance

The growing body of knowledge on psychological safety in the workplace has flourished in recent years, as organizations devote more resources and time to deepen the understanding of the factor role in driving effectiveness. In order to investigate the psychological safety implications in the workplace, Frazier et al. (2017) conducted a meta-analysis drawing from prominent theoretical and empirical works, namely, 136 independent samples representing over 22,000 individuals and nearly 5,000 groups. They have illustrated that psychological safety influences various types of organizational citizenship behaviors and performance. Psychological safety is one of the key factors contributing to better performance of organizations (Schein and Bennis, 1965). By positively influencing employees’ attitude, it can lead to the necessary behavioral modifications making employees inclined to change to better when they feel safe enough to express their opinions. Psychological safety climate impacts employee job design perception and reduces depression and exhaustion from work (Idris et al., 2014). Psychological safety positively influences the job involvement of employees (Brown and Leigh, 1996) and stimulates work engagement (May et al., 2004), which in turn are associated with work effort and performance.

Improving employee’s skills to prepare for future challenges can seriously contribute to the perception of safety and help to deal with uncertainty. Acquiring certain social and professional training and expertise may help to induce psychological safety, as self-efficacy, and performance improve over time, concern about losing job reduces. In line with this, Airila et al. (2014) found that work and personal resources, including self-esteem, are associated with time investment, e.g., investing time into one’s work is related to increased job performance. In addition, enhancing job abilities and increasing work performance is an asset ensuring psychological safety. In line with COR theory, feedback for performance, social support from co-workers and supervisors act as job resources predicting work engagement and performance output (Costa et al., 2015). When there is a safe psychological climate employee put more focus and resources to improve their performance. In a psychologically safe climate employees also report more job resources and more endurable job demands, which lead to a high level of work engagement (Dollard and Bakker, 2010). A survey conducted on data collected from 73 patient-centered healthcare teams confirmed a high level of psychological safety could be a significant predictor of job performance (Kessel et al., 2012). Employees’ sense of psychological safety is linked to feelings of vitality, which result in engaging in creative work (Kark and Carmeli, 2009). Another way psychological safety influences performance at work is by initiating the knowledge-sharing process. Employees with high levels of psychological safety are more likely to be involved in debates and discussions, which stimulate job performance. Healthy debate and the free exchange of opinions guarantee clear expectations employees have from each other (Csikszentmihalyi, 2003). Active participation of all employees in group discussions contributes to higher levels of psychological safety (Randall et al., 1999) and better work results.

Conversely, according to COR, resource loss was found to be more intensive as opposed to resource gain, and even small losses accumulated over time leading to a significant strain. Speaking in biological terms, the fear of losing self-continuity is evolutionarily interpreted as a threat to survival. Stress is a response to a threatening work environment or a change diminishing employees’ resources (Hobfoll, 1989). The conditions that elicit stress are associated with endangering one’s marriage, tenure or health (Oren and Levin, 2017). To ensure the psychological safety, one must exert behaviors not only to protect from resource loss, but even this conservation requires additional resource output. Furthermore, according to some studies, prolonged exhaustion results in defensive, aggressive and irrational behavior (Hobfoll et al., 2018). For instance, psychological distress, e.g., the lack of psychological safety can go as far as to lead to an increase in occupational accident (Siu et al., 2004).

Some research suggests there is a looping effect, namely, an exhausted supervisor may engage in abusive behaviors toward his subordinates by either bullying them or withholding essential resources from them, making a working environment hostile and thus diminishing psychological safety (Cooper et al., 2004; Walter et al., 2015; Lam et al., 2017). Consequently, resource losses in some individuals may trigger resource losses in others, and this depletion of psychological capital induces general distress. Psychological safety also shapes the ways employees deal with and manage conflicts, resulting in benefits for the organization. If a safe climate has been established, workers feel secure and share openly their “creative but potentially embarrassing or controversial ideas” (Bradley et al., 2012). Moreover, a sense of psychological safety makes employees express ideas to higher positioned employees (Avey et al., 2010), and sharing of ideas and knowledge has a significant positive impact on work and team performance (Mesmer-Magnus and DeChurch, 2009; Obrenovic et al., 2015). Crossover surveys revealed that certain psychological states and affection as stress, anxiety, burnout and work-family conflict can transfer from one employee to another (Westman, 2001). Likewise, the spill-over effect is also applicable to the spread of positive emotions. This finding is fundamentally relevant for occupational psychology since it implies it is possible to induce psychological safety by promoting a positive working environment and supplying employees with a set of available resources (brainstorming sessions, know-how exchange, team building and flexible working hours). In effect, the exchange of resources and intellectual and social capital across teams and organizations adds to building the resilience necessary to ensure psychological safety. Psychological resources such as optimism and resiliency are related to increased work-life balance (Siu, 2013). Overall, findings point to the functional relationship between health preservation and increased work performance.

Accordingly, we derive the following hypothesis:

**Hypothesis 3:** Psychological safety impacts job performance.

### Mediating Effects

In a mediation model, work-family conflict impacts job performance via psychological well-being and psychological safety. The above reviewed literature using the COR theory has built a strong justification for the mediating model, supporting individual relationships between variables of the model. COR theoretical framework application on the interaction of work-family conflict in a context of a common resource-exchange economy (Hobfoll et al., 2018) is applied to better understand the mediating effects. According to COR theory, work and family are a reservoir of resources, where a loss or a gain in one domain affects the state of the other (Hobfoll, 2002), impacts personal burnout, distress symptoms (Noor, 2002), and employee well-being (Ibrahim et al., 2009; Fotiadis et al., 2019). Negative work-family interaction decreases well-being due to increased psychological strain and diminished mental resources (Grandey and Cropanzano, 1999; Voydanoff, 2002; Eby et al., 2005). A sense of control over work and family activities has positive implication for psychological well-being (Fitzpatrick et al., 2012). Work-family conflict, as a result of the inability individuals to handle the responsibilities of their family life (Frone and Cooper, 1992) impact on job performance is assessed in the mediating model. Such conflict translates to the psychological states of employees, which have a stronger impact on the job performance. Prior studies have also identified significance of work-family conflict for job performance (Frone et al., 1992; Netemeyer et al., 2005), which is an assumption of the valid mediating model. The mediator variables of psychological well-being and psychological safety explain how work-family conflict affects job performance. Thus, we propose:

**Hypothesis 4:** There is a mediating effect of psychological well-being and psychological safety between work-family conflict and job performance.

## Materials and Methods

### Participants and Procedure

A survey strategy was employed, and a questionnaire was distributed to a sample of individuals employed in a company in Bahrain. The company is an international manufacturing and service company serving the gas and oil industry sector in Bahrain. The original scales were translated from the English language to Arabic language and back, to ensure meaning has not been lost in the translation process. This questionnaire used closed-ended questions to collect demographic data and a five-point Likert-scale to measure the variables of the research model. The scale items were evaluated from Strongly agree (5) to Strongly disagree (1), and from Much more than usual (5) to Much less than usual (1). All the data collected is based on self-rated responses of employees. Supervisors were not participating in the study and have not been asked to evaluate employee performance as a part of the questionnaire. The data collection took place from June 2018 to August 2018. All the participants of the study were informed of the investigation aims and provided their verbal consent to participate in the survey. A self-report questionnaire was administered to 500 individuals, out of which 359 participated in the study. Only fully filled questionnaires were taken into consideration for further analysis. After the elimination of questionnaires with missing values, the final sample consisting of 277 employees, was investigated in detail, 217 men, and 60 women, respectively. Out of the participants who were analyzed, 28.5% of employees were between the ages of 35 and 44, and 69.4% of the participants were between 24 and 35 years old. Only 2.1% of the participants were over 55 years old.

Participants with less than 5 years of working experience constituted 41.5% of the analyzed sample, and 34.2% of participants had 6–10 years of experience. Most of the respondents had a bachelor’s degree (59.1%), whereas 20.1% of the respondents had completed high school. The rest were holding a master’s degree (20.8%). As for the present job responsibilities in their respective company, most of the employees were employed in the Manufacturing-Operations department (22.1%) and in Marketing-Sales (20.7%). Human resources (11.7%) and Accounting (9.5%) were also among the highest represented departments in this study. Less than 5% of the respondents worked in the Finance department.

### Measurements

#### Psychological Well-Being and Psychological Safety

Psychological well-being and psychological safety were assessed using the twelve-item General Health Questionnaire (GHQ-12) developed by Goldberg and Williams (1988). GHQ-12 is a validated instrument that measures the psychological well-being and psychological safety of employees. Commonly the instrument is used to detect the symptoms of ailments, such as non-psychotic psychiatric disorders. Example items measuring psychological well-being are “Able to concentrate,” “Capable of making decisions,” and “Constantly under strain.” Psychological safety example items include items “Feeling unhappy and depressed” and “Losing confidence in yourself.” Cronbach’s alpha was used to evaluate the internal consistency of the scale, exhibiting an adequate level of 0.799 for psychological well-being and 0.812 for psychological safety, respectively.

#### Work-Family Conflict

A nine-item scale was adopted from Carlson et al. (2000) measuring work-to-family conflicts. Example items are “My work keeps me from my family activities more than I would like,” and “I have to miss family activities due to the amount of time I must spend on work responsibilities.” Scale exhibited adequate reliability of 0.889.

#### Job Performance

Job performance scale measuring the in-role performance of employees was adopted from Williams and Anderson (1991). The scale consists of seven items. The items were worded so that employees could assess their in-role performance. For instance, “Adequately complete assigned duties” and “Fulfill responsibilities specified in the job description.” Two items were reverse coded: “Neglect aspects of the job I am obligated to perform” and “Fail to perform essential duties.” Cronbach’s alpha value of the job performance scale was 0.781, indicating internal consistency.

## Statistical Analysis

**FIGURE 1:**
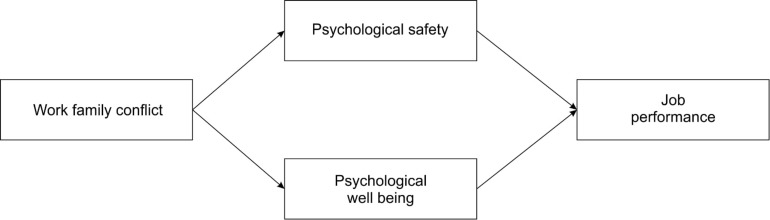
Research model of the study.

The research model was structured to explain the influence of work-family conflict on psychological well-being, and psychological safety, and the impacts of psychological well-being, and psychological safety on job performance, as well as their mediating effects (see Figure 1). Analysis was performed by using SEM software AMOS version 23. The missing values for 82 respondents were eliminated, and in the end, statistical indicators for the sample of 277 respondents were analyzed. Descriptive statistics for each item and summary variables were calculated. Means, SD, skewness, and kurtosis indicators for variables are exhibited in Table 1. Furthermore, correlation analysis has been performed with results presented in Table 2. The variables are all appropriately correlated, fitting to the existing theory. The goodness of fit indices (χ^2^/*df*, GFI, SRMR, RMSEA, CFI, AGFI, GFI, NFI, and RFI) were used to evaluate model goodness of fit. The values of indices are summarized in Table 3. Confirmatory Factor Analysis with Maximum likelihood estimation was performed for each scale, and for the observed models. Common Latent Factor (CLF) method, which uses a CLF to capture the common variance among all observed variables in the model was utilized. In the structural model testing with SEM relationships between work-family conflict, psychological well-being, psychological safety, and job performance were examined. As part of the path analysis standardized parameter estimates, standard errors, and *p*-values for the structural model were calculated. In order to confirm the significance of the relationship *p*-value with the lowest significant point of 0.05 and C.R. representing the critical value were utilized. The judgment of a good fit of the model when C.R. value is equal or bigger than 1.96 or in the opposite direction – when it is equal or lower than (−1.96). Bootstrap approach to calculate 95% of the bias-corrected bootstrap confidence interval (CI) of the unstandardized indirect effects was applied. Hayes (2009) procedures were followed to formulate the mediating hypotheses. A mediation has been assumed implying that independent variable of work-family conflict impact on the dependent variable of job performance has been diminished after the mediator variables have been introduced.

**TABLE 1:** Descriptive statistics.

Statistic	*N*	Minimum	Maximum	Mean	SD	Skewness	Kurtosis
Psych.Well	277	1.00	3.00	2.0377	0.54250	–0.465	0.600
Psych.Safe	277	1.00	4.00	2.5833	0.60400	–0.332	–0.059
W.F.Conf	277	1.00	5.00	2.9601	0.59327	–0.060	0.914
Job.Perf	277	2.00	4.71	3.3975	0.43375	–0.016	0.377
Valid *N* (listwise)	277						

**TABLE 2:** Pearson correlation analysis.

	Psy. well-being	Psy. safety	Work-family conf.	Job performance
Psy. well-being	1	–0.037	–0.103	0.318**
Psy. safety	–0.037	1	0.158*	−0.149*
Work-family conf.	–0.103	0.158*	1	−0.227**
Job performance	0.318**	−0.149*	−0.227**	1

* Correlation is significant at the 0.05 level (2-tailed). ** Correlation is significant at the 0.01 level (2-tailed).

**TABLE 3:** Summary of model fit indices.

	χ^2^/*df*	SRMR	RMSEA	CFI	AGFI	GFI	NFI	RFI
Measurement model	1.886	0.066	0.057	0.913	0.893	0.925	0.836	0.791
Hypothesized structural model	57.038	0.239	0.451	0.559	0.274	0.855	0.562	−0.315
Improved structural model	0.831	0.017	0.010	1.000	0.985	0.998	0.997	0.981
Thresholds (Hu and Bentler, 1999; Hoyle, 2000; Thompson, 2005; Kline, 2011)	<3	<0.06/10	<0.08	>0.90/95	>0.80	>0.95	>0.90	>0.90

*χ^2^/df = normed chi-square statistic; GFI, goodness-of-fit index; RMR, root-mean-square residual; RMSEA, root mean square error of approximation; NFI, normed fit index; TLI, Tucker–Lewis index; CFI, comparative fit index.*

## Results

### Path Analysis and Confirmatory Factor Analysis Results

Firstly, the confirmatory factor analysis has been performed for individual scales. Factor loadings were examined with items of loadings lesser than 0.55 as recommended by Fornell and Larcker (1981a, b) were eliminated, unless deemed essential. The bifactorial model achieved a better fit than the single-factor model for Psychological well-being and Psychological safety (χ^2^/*df* = 2.947; CFI = 0.658; SRMR = 0.105; RMSEA = 0.084). For the Work-family conflict scale a three-factor model exhibited a suitable fit, whereas for job performance scale bifactorial model showed appropriate fit (χ^2^/*df* = 2.947; CFI = 0.658; SRMR = 0.105; RMSEA = 0.084). Next, the four-factor model was hypothesized to be confirmed in measurement model testing (Figure 1).

**FIGURE 2:**
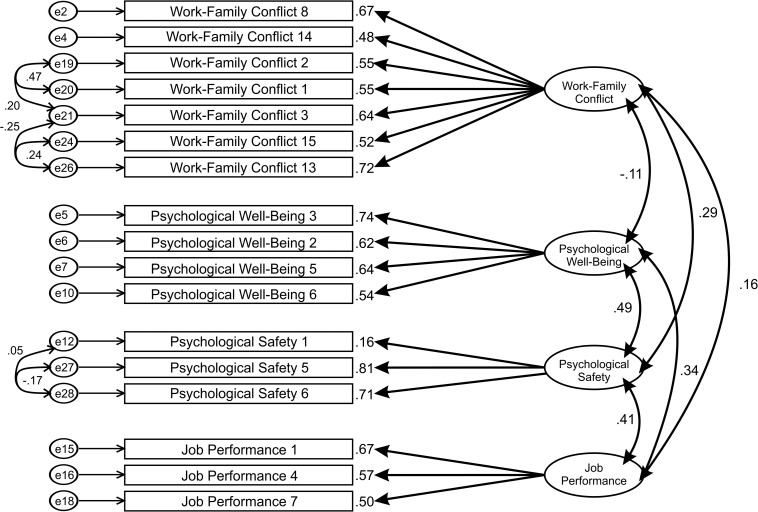
Measurement model.

The confirmatory factor analysis revealed that the measurement model did not achieve a good model fit (χ^2^/*df* = 2.897; CFI = 0.686; SRMR = 0.103; RMSEA = 0.083). Accordingly, the model was improved by adding covariances based on modification indices and by deleting several items based on standardized residual covariances. Once again, the model was tested, and the measurement model achieved an acceptable model fit (χ^2^/*df* = 2.494; CFI = 0.832; SRMR = 0.091; RMSEA = 0.074) (Figure 2). Therefore, a subsequent CFA has been performed resulting in an excellent model fit (χ^2^/*df* = 2.947; CFI = 0.913; SRMR = 0.66; RMSEA = 0.057). χ^2^/*df* ratio was below the recommended value of three (Hair et al., 2013). The standardized root mean square (SRMR = 0.063) was less than.08 as recommended, which indicates a good model fit (Byrne, 2010). Also, the value root mean square error of Approximation was less than.08 (RMSEA = 0.057), while the value of the Comparative Fit Index was above 0.9 (CFI = 0.913). Consequently, it is indicated that the model is a representation of a good model fit (Hu and Bentler, 1999; Byrne, 2010). Unstandardized and standardized parameter estimates obtained in this research are provided in Table 4.

**TABLE 4:** Standardized parameter estimates, standard errors, and p values for the measurement model.

	SRW	URW	S.E.	C.R.	*p*
Work.Fam.Conf8 ← WFC	0.668	1	–	–	–
Work.Fam.Conf14 ← WFC	0.484	0.726	0.107	6.803	***
Psycho.Well.B3 ← WELL.B	0.739	1	–	–	–
Psycho.Well.B2 ← WELL.B	0.616	0.864	0.107	8.088	***
Psycho.Well.B5 ← WELL.B	0.639	0.862	0.104	8.284	***
Psycho.Well.B6 ← WELL.B	0.538	0.746	0.102	7.293	***
Psycho.Safety1 ← SAFETY	0.161	1	–	–	–
Job.Perform1 ← JOB.P	0.672	1	–	–	–
Job.Perform4 ← JOB.P	0.575	0.987	0.18	5.481	***
Job.Perform7 ← JOB.P	0.501	0.807	0.153	5.272	***
Psycho.Safety5 ← SAFETY	0.814	4.435	3.145	1.41	0.158
Psycho.Safety6 ← SAFETY	0.71	4.095	2.899	1.412	0.158
Work.Fam.Conf2 ← WFC	0.547	0.84	0.114	7.352	***
Work.Fam.Conf1 ← WFC	0.546	0.909	0.12	7.552	***
Work.Fam.Conf3 ← WFC	0.644	0.99	0.126	7.88	***
Work.Fam.Conf15 ← WFC	0.523	0.806	0.118	6.807	***
Work.Fam.Conf13 ← WFC	0.72	1.087	0.13	8.367	***

*** p value <0.001; SRW, standardized regression weights; URW, unstandardized regression weights; C.R., critical value.

**FIGURE 3:**
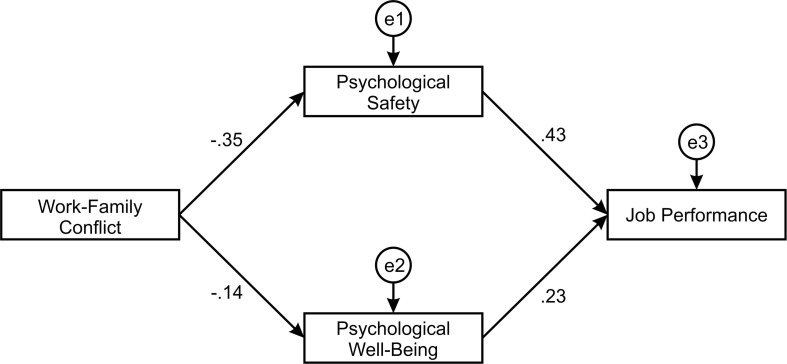
Hypothesized structural model.

The factors confirmed during the Confirmatory Factor Analysis were used in the structural model. Firstly, the hypothesized model (Figure 3) was tested for model fit. This structural model has not achieved an adequate model fit according to the obtained model fit indices given in Table 2 (χ^2^/*df* = 57.038; CFI = 0.559; SRMR = 0.239; RMSEA = 0.451). A common method bias test was conducted. The zero-constrained method was utilized. The unconstrained common method factor model was compared to the fully constrained common method factor model. The chi-squared test was significant (χ^2^ = 34.1, *df* = 17, *p* = 0.008). According to the result, there was substantial shared variance. Therefore, the imputation of factor score was done, and the obtained factors were accounted for the shared variance explained by a CLF.

**FIGURE 4:**
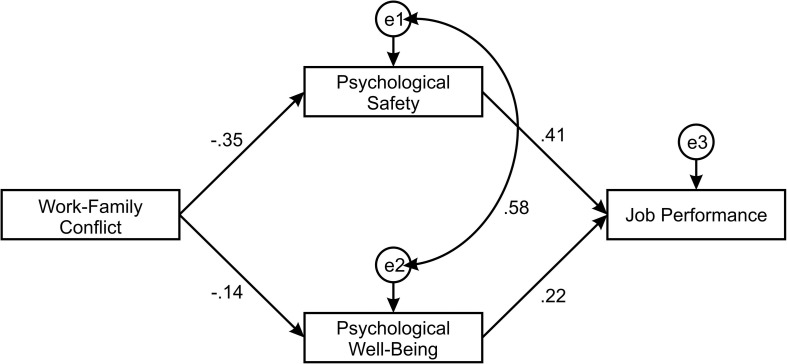
Improved structural model.

In order to improve the hypothesized model, one covariance was added between error terms related to the Psychological safety and Psychological well-being (Figure 4). Thus, the structural model achieved a good model fit according to the model fit indices given in Table 2 (χ^2^/*df* = 0.831; CFI = 1.000; SRMR = 0.017; RMSEA = 0.010).

Standardized parameter estimates, standard errors, and p values for the structural model were calculated as a part of path analysis in AMOS. According to the results showed in Table 5, all direct paths in the structural model were statistically significant. Work-family conflict had a negative direct effect on Psychological safety (β = −0.351; p < 0.01) and negative direct effect on Psychological well-being (β = −0.137; p < 0.05). Thus Hypothesis 1a and Hypothesis 1b were accepted. A significant relationship between Psychological well-being and Job performance (β = 0.218; p < 0.001) has been identified, resulting in acceptance of Hypothesis 2. As for the influence of Psychological safety on Job performance a significant impact has been found (β = 0.401; p < 0.001), with Hypothesis 3 being accepted as well.

**TABLE 5:** Standardized parameter estimates, standard errors, and p values for the structural model.

Paths	SRW	S.E.	C.R.	*p*
PSYCH.SAFE ← WORK.FAM.CON	–0.351	0.013	–6.229	***
PSYCH.WELL ← WORK.FAM.CON	–0.137	0.048	–2.289	0.022
JOB.PERF ← PSYCH.SAFE	0.406	0.193	6.660	***
JOB.PERF ← PSYCH.WELL	0.218	0.055	3.547	***

*** p-value <0.001; SRW, standardized regression weights; S.E., standard error; C.R., critical value.

### Mediating Effects

To determine the mediating effects as the first step the impact of work-family conflict on job performance has been established as indicated in Table 6. Additionally, indirect effects were tested, and obtained results indicated that Work-family conflict had an indirect influence on Job performance when Psychological safety was a mediator. The direction of relationship is negative with the value of (β = −0.183) and high level of significance confirmed with the bootstrap test (p = 0.004). On the other hand, the indirect relationship between Work-family conflict and Job performance is reducing when Psychological well-being serves as a mediator. The value of their relationships equals to (β = −0.059). Even though the direction of the indirect relationship is also negative, the significance value is barely over the threshold of p < 0.05 (p = 0.04). According to the mediation results the Hypothesis 4 is accepted.

**TABLE 6:** Direct, indirect, and total effects.

Pathway	Standardized direct effect	Standardized indirect effect	Standardized total effect	Evidence of
Job Performance ← Work.Fam.Conflict	–0.031	−0.183*	−0.225*	Indirect negative, significant effect (Psych. Safety as mediator)
Job Performance ← Work.Fam.Conflict	−0.155*	−0.059*	−0.225*	Direct negative significant effect (Psych. Well Being as mediator)

*Correlation is significant at the 0.05 level (2-tailed).

## Discussion

In this empirical study, the influence of work family conflict on psychological well-being and psychological safety was assessed. We posited that the two factors mediate the relationship between job performance and work-family conflict. The results of the paper indicate that there is a relationship between work-family conflict and psychological well-being, and between work-family conflict and psychological safety. The findings of the study are in line with past studies that analyzed the influence of work-family conflict on psychological well-being (Kossek and Ozeki, 1998; Ford et al., 2007). Consistent with the previous studies (Grzywacz and Bass, 2003; Aryee et al., 2005; Voydanoff, 2005; Kinnunen et al., 2006), we found that the impact of work-family conflict on well-being demonstrates that employees are more likely to be stressed with negative work-family interaction. The negative impact of psychological well-being can be demonstrated by impacting physical, behavioral and cognitive-affective parts of their daily life (Allen et al., 2000). The findings of Siu et al. (2005), suggest there is a relationship between work-family stressors, job satisfaction and well-being.

We also investigated to which degree work-family conflict influences psychological safety. The study findings demonstrate the impact of work-family conflict on psychological safety, which is consistent with the previous studies (Randall et al., 1999; May et al., 2004). The results of the analysis reveal there is a negative effect on the psychological safety of the employees. At a professional level, an organization can contribute to the psychological safety of employees by preventing conflicts from occurring, and by providing counseling to employees for family or work-related issues that emerge. When the conflicts occur, organizations can suffer severe damage to their performance, since employees’ performance is mediated by the perception of psychological safety (Johnson et al., 2005). Organizational stimuli generating a positive organizational climate can help facilitate a nurturing and worry-free environment. The findings of the research conducted by Carmeli and Gittell (2009) reported that work relationships which include shared goals, knowledge, and mutual respect stimulate psychological safety, and enable the members of the team to engage in the learning process. Mansour and Tremblay (2016) discovered that when employees believe that organizations care for their personal life, they spend more and better-quality time at home, which can decrease the family burden and reduce stress. Conversely, work overload, exhaustion, and lack of freedom at the workplace may cause an individual to convey job-related frustration in a work or family setting. Recent research attempts focused on investigating the repercussions of a toxic working environment entailing a feedback effect and causing psychologically exhausted employees to bring negative emotions back to the workplace. Work stress was central to organizational research as it was harmful to employees and organizations. Role conflict, ambiguity, situational constraints, and exhaustion reduced positive job outcomes resulting in lower task performance (Cooper et al., 2001). Emotional dissatisfaction, inter-role and intra-role distress reflect on work and family domains, resulting in counterproductive behavior, toward co-workers, and organizations (Bennett and Robinson, 2000; Yu et al., 2019). There is a reciprocal relationship between organizational constraints and subsequent counter-productive work (CWB) behavior, resulting in a vicious cycle where CWB affects actors and targets (Meier and Spector, 2013). Bolger et al. (1989) described this as an interpersonal process where job stress experienced by an individual affects their social network. For instance, when a problem occurs in the family domain, more resources are brought in by the individual to fix it, resulting in higher levels of exhaustion and burnout. This manifests in a work sphere as a decreased self-regulation capability and a lack of self-control resources to manage inappropriate actions against the organization and increased aggressiveness toward team members (Savickas, 2002, 2005). This is especially evident among supervisors and subordinates. Lam et al. (2017) reported on evaluations for measuring the levels of aggression at the workplace, whereby bullying is defined not only as an abuse but also as a withholding of useful information or denying a constructive positive feedback (Moss et al., 2003; Tepper et al., 2006; Whitman et al., 2014). The intervention was proposed by Hammer et al. (2009) where supportive policies were introduced to help employees achieve their goals and manage their work and family roles more effectively. Organization thereby acts as an additional resource provider in order to improve overall work climate, since according to the crossover model, cognitive and emotional capital transfers among actors within the same social and organizational contexts. The aim is to restrain the consequences of mobbing and other abusive, defamatory and malignant behaviors. Conversely to the fostering and exchange of positive capital building upon the resiliency, its depletion was revealed to just add to team members’ distress and vulnerability (Li et al., 2016). The effects of the negative working climate on the individual are already well established, and what warrants investigation is how such negative factors impact working climate. The lack of social, emotional, and organizational support can further feed into this negative mechanism. Thus, it would be beneficial for organizations to tap into and get more involved not solely in the issues regarding the pressing emergence of taunting behavior, but also the underlying motivations for lashing out. Wallace et al. (2009) hypothesized the differential relationship between challenge and hindrance stressors, demonstrating the existence of a negative association between hindrance and role-based performance. Implications clearly state organizational support in the workplace could be favorable in eliminating stressors.

The study findings show that psychological safety and psychological well-being influence job performance. The findings are in accordance with evidence generated by previous research on psychological health (Wright and Cropanzano, 2000; Bakker and Schaufeli, 2008; Avey et al., 2010; Fotiadis et al., 2019). A study by Cartwright and Cooper (2008) also reported that people with high levels of psychological well-being are healthier and more productive at work. Psychological well-being can be influenced by health problems, work-related problems, and family problems. We hypothesized that when the psychological well-being of employees is unsatisfactory, the job performance will decrease accordingly. Furthermore, the mediating effect of psychological well-being has been identified. To support our hypotheses we have utilized COR theory, and the current findings can be interpreted using COR theory in a sense that employees with lower psychological well-being have lower work performance due to depleted psychological resources, such as pessimistic attitude and being less resilient. Negative emotions arising from work-family conflict can harm the psychological well-being of employees, manifesting in negative attitude toward the completion of one’s duties and decreased employee effectiveness.

In line with the theoretical and practical assumptions of Schein and Bennis (1965) and Avey et al. (2010), this study suggests a working environment characterized by psychological safety is essential for individuals to feel secure and thus capable of exchanging ideas and being productive at work. Employees with a higher degree of freedom are likely to share knowledge and innovate, thus stimulating organizational development. Additionally, psychological safety leads to higher job performance as employees who feel secure are more engaged in their work. Namely, when ideas and opinions of employees are considered and accepted, employees tend to work harder to succeed in their jobs. The findings of Idris et al. (2014) suggested that psychological safety climate had a considerable impact on employee perceptions of job design, and it reduced psychological problems of exhaustion and depression of employees. Social support, communication and feedback from co-workers also have a positive influence on job performance (Costa et al., 2015). In a safe psychological climate, employees put more focus and resources to improve their performance. Employees who are unable to express themselves due to the high level of interpersonal risk stemming from the work environment are less likely to collaborate and will have diminished job performance.

Morgeson et al. (2017) research on the further development of COR theory show that managerial and organizational, as well as social support in the view of resources provided, may be culturally dependent. The spillover effect of work-family conflict is found to be different in societies with a strong tradition of marital commitment (Liu et al., 2016). The current empirical study was conducted in Bahrain, an Islamic religious background. A study conducted by Hofstede (2003) shows that resources are conceived and provided differently relative to the culture in question and whether it is individualistic or collectivistic. In individualistic societies, organizational policies are directed to provide encouragement and training with the aim of preserving individual resources through therapy, such as physical and psychological resources. In collectivistic cultures, more attention is devoted to group benefits as they are assumed to impact the integrated whole; thus the aim is to create a caring and safe environment throughout social activity, stressing the importance of preserving social harmony. Based on the results obtained in this study, we conclude that organizations should promote a safe working environment where employees can freely interact, as this will lead to an increase in their job performance.

### Implications of the Study

The present study suggests there is a relationship between work-family conflict and psychological well-being and safety, with the ultimate impact on job performance. This notion represents a significant contribution as the theoretical gaps are successfully addressed, and the field of organizational psychology is enriched. This study is important since it detaches from the prior researches focused on observing the repercussions of work-family conflict in workers’ well-being, and centers on the analysis of job performance instead. Innovative points of this study translate to practical implications for managers and leaders, suggesting they must not only inspire and foster a healthy friendly environment that will motivate employees to apply energy and reach full potential thus increasing job performance, but also alleviate work-family conflict that can lead to negative organization behaviors. Fulfilling company objectives strongly depends on the performance of employees, and therefore it is essential to create favorable working conditions that stimulate employees to increase their job performance. However, in many cases, organizations fail to understand the underlying reasons behind employee effectiveness and job performance. In this regard, understanding the antecedents of work-family conflict allows the organizations to create favorable conditions and foster beneficial mental states of employees. Employees experience less work-family conflicts and are more effective in organizations with influential organizational culture (Bakker and Schaufeli, 2008). In such organizational cultures, the likelihood of work-family conflict is minimized, and the company could benefit from employee work engagement. Reflecting on the available evidence, we find that the well-being of the individuals triggers feedback to their work environment, thus influencing further their demands and resources, and this influence should be observed by organizational leaders, and changes should be initiated accordingly.

Consequently, organizations should focus on building a safe and stable organizational culture. Job performance is determined by psychological well-being and psychological safety. Accordingly, managers should pay more attention to improving those two states, mainly by preventing work-family conflicts. Reducing the occurrence of work-family conflict and mitigating its impact can bring favorable results for the productivity of employees. Organizations should identify and implement strategies to reduce work-family conflict occurrence and enhance work-life balance. A flexible schedule and decreasing the intensity of work are viable options organizations ought to consider. Additionally, introducing novel communication tools built on innovative technologies could have a significant impact on relieving employees from work stress (Cascio and Montealegre, 2016). Organizations need to take into consideration not only work duties but also family duties the employees have. Nowadays, a limited number of organizations take necessary employee retention and development measures. In this regard, organizations could offer a variety of training and educational sessions enabling employees to grow personally and professionally. It is also essential to consider different methods that are used to manage work-family conflict. Literature suggests work-family conflicts can be managed by focusing on the problem itself or on emotions while coping with the conflict (Rapoport et al., 2002). Another contribution of the study is that it was conducted among employees from Islamic culture, a country of Bahrain. Up to date, the majority of the studies were conducted in Western countries.

### Prospective Research and Limitations

There are several study limitations that should be considered. First, the current study was cross-sectional in nature. Future studies should collect data at different points in time to evaluate how job performance changes with work-family conflict resolution, as well as with improved psychological well-being and safety. In our research, we approached work-family conflict from a perspective of work role interference with the family role. Future studies can explore cases when individuals have multiple roles to execute but cannot decide which one to pursue. Another limitation is that the convenience sampling method was used. The study was conducted on a sample of employees from a company in Bahrain. Therefore, there is a question of whether the study is generalizable. The current study can be replicated, and future studies need to validate the findings of the study, both on the sample of other companies in Bahrain, and in other countries. The sample also consisted of more men than women, thus future studies should explore gender differences and work-family conflict relationships and identify whether males or females are more susceptible to stress when executing multiple roles.

Additionally, the impact of specific elements of work-family conflicts, such as time, energy, and behavior on job performance warrants more investigation. A limited number of studies explore the specific influence of those elements on job performance. The impacts of work-family conflict, psychological well-being, and safety on the health of employees should be examined too. Furthermore, an investigation on the impact of psychological well-being on the creation of negative emotions in humans is warranted, particularly the type of emotion that arises due to the work-family conflict. Finally, self-reported measures were used to evaluate the variables of the model, including the job performance. Future studies could potentially use supervisors’ observations and management evaluation for the employees’ performance to validate the research findings of our study.

### Conclusion

The current study proposes that work-family conflict is a significant predecessor of job performance. Understanding the nature of the work-family conflict, being aware of its causes and the ways to prevent it is crucial for ensuring the success of organizations. The study confirms the connection between the work-family conflict and psychological well-being as well as psychological safety. Poor job performance is associated with a lack of psychological safety and psychological well-being of employees. Psychological safety and well-being are mediators in the relationship between work-family conflict and job performance. The growing body of knowledge in organizational psychology field confirms that work-family interaction and psychological health is a crucial topic of interest for scholars and experts.

## Data Availability Statement

The datasets are available on request: The raw data supporting the conclusions of this article will be made available by the authors, without undue reservation, to any qualified researcher.

## Ethics Statement

This research was conducted in accordance with the highest research standards and approved by the Ethics Committee of Jiangsu University. Before starting the questionnaire, participants agreed to informed consent that provided the general research aims. They were informed that participation was voluntary and that their responses are anonymous and treated confidentially. Moreover, they were provided with contact information of the researchers and the ethical committee.

## Author Contributions

BO conceived the idea, contributed to the design of the study, was involved in all steps of the research process, and wrote a first set-up and draft of the manuscript. DJ researched the statistical methods, contributed to the design of the study, data acquisition, result interpretation, and drafted the manuscript. AK contributed to the design of the study, data collection and adjustments, and wrote additions. MK made a substantial, direct and intellectual contribution to the work, edited the manuscript, and approved it for publication. All authors approved the manuscript and agree to be accountable for all aspects of the work.

## Conflict of Interest

The authors declare that the research was conducted in the absence of any commercial or financial relationships that could be construed as a potential conflict of interest.
